# The Multiple Regulatory Relationship Between RNA-Chaperone Hfq and the Second Messenger c-di-GMP

**DOI:** 10.3389/fmicb.2021.689619

**Published:** 2021-07-14

**Authors:** Yang Fu, Zhaoqing Yu, Li Zhu, Zhou Li, Wen Yin, Xiaodong Shang, Shan-Ho Chou, Qi Tan, Jin He

**Affiliations:** ^1^National Engineering Research Center of Edible Fungi, Institute of Edible Fungi, Shanghai Academy of Agricultural Sciences, Shanghai, China; ^2^State Key Laboratory of Agricultural Microbiology, College of Life Science and Technology, Huazhong Agricultural University, Wuhan, China

**Keywords:** RNA-chaperone Hfq, c-di-GMP, small RNA, c-di-GMP metabolic enzymes, biofilm, motility, virulence, regulatory relationship

## Abstract

RNA chaperone protein Hfq is an important post-transcriptional regulator in bacteria, while c-di-GMP is a second messenger signaling molecule widely distributed in bacteria. Both factors have been found to play key roles in post-transcriptional regulation and signal transduction pathways, respectively. Intriguingly, the two factors show some common aspects in the regulation of certain physiological functions such as bacterial motility, biofilm formation, pathogenicity and so on. Therefore, there may be regulatory relationship between Hfq and c-di-GMP. For example, Hfq can directly regulate the activity of c-di-GMP metabolic enzymes or alter the c-di-GMP level through other systems, while c-di-GMP can indirectly enhance or inhibit the *hfq* gene expression through intermediate factors. In this article, after briefly introducing the Hfq and c-di-GMP regulatory systems, we will focus on the direct and indirect regulation reported between Hfq and c-di-GMP, aiming to compare and link the two regulatory systems to further study the complicated physiological and metabolic systems of bacteria, and to lay a solid foundation for drawing a more complete global regulatory network.

## Introduction

Bacteria are single-celled organisms without complex tissues and systems, but they often live in highly variable physical and chemical environments in nature. The reason why bacteria can adapt to harsh ecological environments and exhibit tenacious vitality is largely due to the pressure exerted by the environment, which forces them to establish effective and diverse signaling response systems in the long-term evolution process to achieve better survival and reproduction. In addition, the potential coordination and regulation between the different systems can also endow bacteria with excellent environmental adaptation and survivability. These regulatory factors include Hfq, an important RNA-chaperone protein, and c-di-GMP, a vital second signaling molecule, both of which are widely distributed in bacteria and may be involved in the regulation of similar physiological activities.

Hfq can associate with various small RNAs (sRNAs) and/or mRNAs to regulate the expression of target genes at the post-transcriptional level (Vogel and Luisi, 2011). Due to the wide variety of sRNAs and mRNAs that can bind Hfq, it has been found that Hfq is involved in numerous complex phenotypes, such as bacterial growth regulation, chemotaxis and motility, biofilm formation, and virulence factor expression. Hfq can be considered as a global post-transcriptional regulator because it regulates many downstream genes that affect numerous phenotypes. C-di-GMP, on the other hand, is a key nucleotide second messenger molecule that has been discovered to be widely distributed in bacteria (Jenal and Malone, 2006; Römling et al., 2013). Under the stimulation of extracellular and intracellular signals, bacteria can change the concentration of intracellular c-di-GMP by altering the activities of various metabolic enzymes. C-di-GMP achieves signal amplification and functional output by binding to various downstream receptors, leading to changes in bacterial phenotypes, such as cell cycle, cell differentiation, motility, biofilm formation, and virulence (Schirmer and Jenal, 2009; Boyd and O’Toole, 2012; Hengge, 2021).

Although Hfq and c-di-GMP play key roles in the post-transcriptional regulation and signal transduction, respectively, and appear to be independent of each other, they still have been reported to be involved in the regulation of several common phenotypes. Hence, we speculate that there may be regulatory relationship between Hfq and c-di-GMP. Therefore, after briefly introducing the importance of Hfq and c-di-GMP regulatory systems, we will focus on the various direct and indirect regulation that have been reported between them. We hope that this review will enrich our understanding on the interactions between the Hfq and c-di-GMP systems and provide a more complete picture of the global communication network between different bacterial regulatory systems.

## RNA Chaperone Hfq

Hfq was first discovered to be an essential host factor required for the RNA synthesis of *Escherichia coli* phage Qβ (Franze de Fernandez et al., 1968), but was later found to be a common RNA chaperone protein in bacteria. Like the Sm and Lsm spliceosome proteins, which are mainly involved in RNA degradation in eukaryotes and archaea, Hfq also belongs to the Sm/Lsm family of RNA-binding proteins (Valentin-Hansen et al., 2004). The secondary structure of various Hfq protein monomers in different bacteria is highly conserved, with all containing a conserved Sm domain for RNA binding (Beich-Frandsen et al., 2011; Vincent et al., 2012; Mura et al., 2013; Santiago-Frangos and Woodson, 2018). We mainly take Hfq in *E. coli* as an example to introduce its structural characteristics. The conserved Sm motif is usually located at the N-terminus of the secondary structure of Hfq monomer, while the C-terminus is flexible among different bacteria species (Vogel and Luisi, 2011; Santiago-Frangos and Woodson, 2018). It has been proven that the N-terminus containing the Sm motif mainly performs the function of RNA binding, but the specific function of the disordered C-terminus is less clear. It is thus speculated that the flexible C-terminus of Hfq may contribute to the diversity of Hfq functions in different bacteria species (Vogel and Luisi, 2011).

Hfq usually exists in the form of a C6 symmetric cyclic homo-hexamer, and forms two asymmetric RNA binding surfaces, namely the proximal and the distal surfaces (Vogel and Luisi, 2011). In the quaternary structure of Hfq hexamer, the surface with the exposed N-terminal α-helix is called the proximal surface, while the opposite side is called the distal surface. Some researchers have also found that the lateral rim of Hfq hexamer can interact with RNA (Ishikawa et al., 2012; Sauer et al., 2012). Hfq thus uses different RNA-binding surfaces to bind various RNAs to identify accurate and specific sequences or motifs on RNA to regulate their functions (Santiago-Frangos and Woodson, 2018).

**FIGURE 1:**
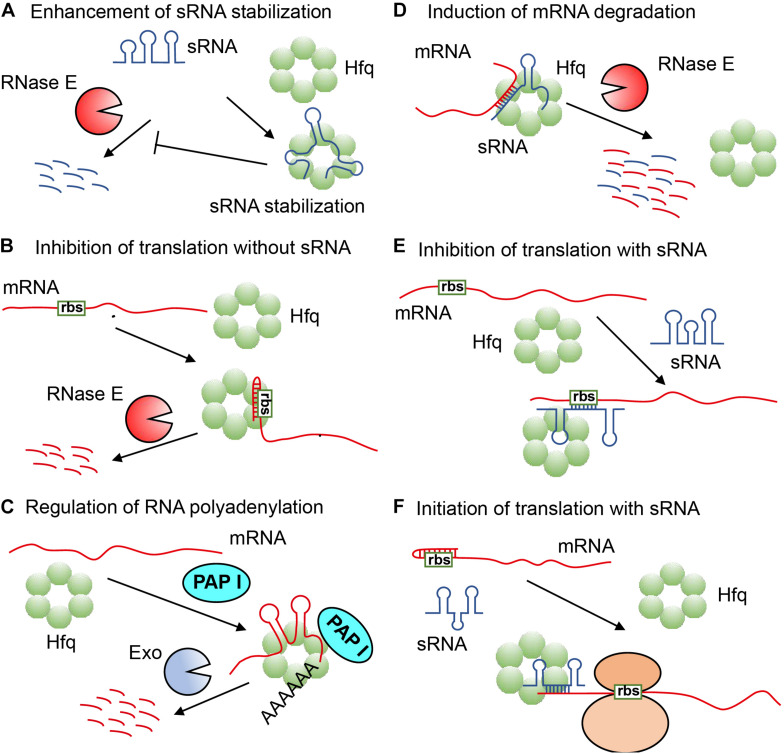
The various regulation mechanisms exhibited by Hfq (Vogel and Luisi, 2011; Dos Santos et al., 2019). **(A)** Hfq refolds sRNA to protect it from ribonuclease cleavage. **(B)** Hfq refolds the leader sequence of mRNA to form a stem-loop structure within the ribosome binding site (rbs) to block the formation of translation initiation complex, and cause mRNA to be rapidly degraded by RNase E. **(C)** Hfq refolds mRNA for polyadenylation by polyA polymerase to promote the degradation of mRNA by an exoribonuclease (Exo). **(D)** Hfq refolds sRNA and mRNA for partial pairing, causing them to be simultaneously degraded by RNase E. **(E)** Hfq refolds sRNA and mRNA for partial pairing within the leader sequence of ribosome binding site to inhibit translation. **(F)** Hfq refolds sRNA and mRNA to expose mRNA ribosome binding site for translation initiation.

**FIGURE 2:**
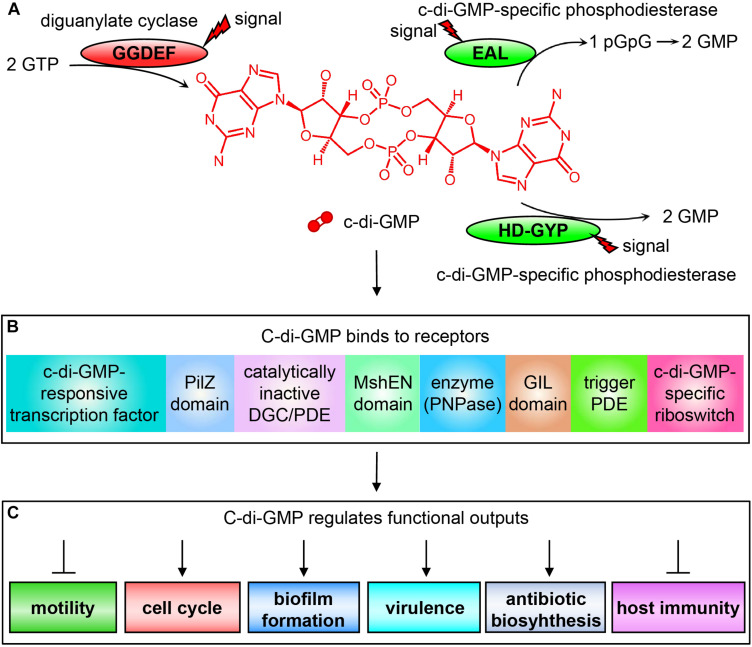
Schematic diagram of c-di-GMP signal transduction system. **(A)** The synthesis and degradation of c-di-GMP depending on the DGCs containing the GGDEF domain and c-di-GMP-specific PDEs containing EAL or HD-GYP domain. **(B)** The c-di-GMP receptors found in bacteria that bind to c-di-GMP for exerting their regulatory functions. **(C)** Different physiological phenotypes of bacteria regulated by c-di-GMP binding to receptors.

**FIGURE 3:**
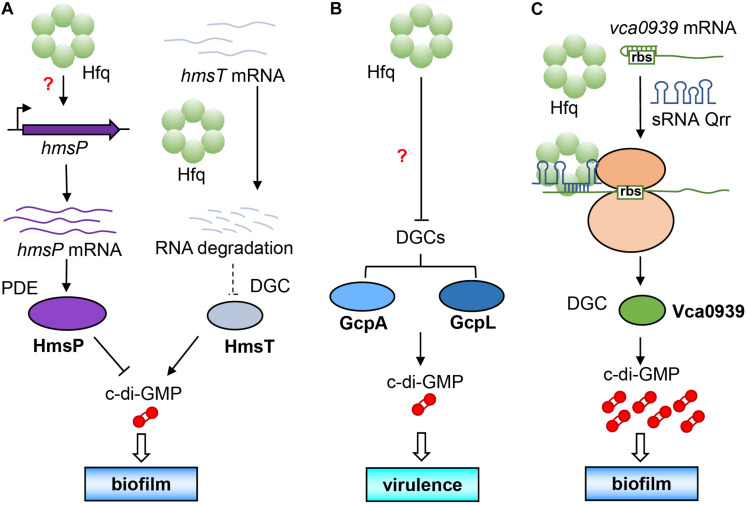
Direct regulation of intracellular c-di-GMP concentration by Hfq. **(A)** Hfq increases the expression of *hmsP* PDE gene at the transcriptional level by an unknown mechanism and reduce the expression of HmsT DGC at the post-transcriptional level, causing a decline of c-di-GMP level to affect its biofilm formation. **(B)** Hfq simultaneously inhibits the expression of two DGCs (GcpA and GcpL) by an unknown mechanism to decrease c-di-GMP biosynthesis, leading to a decreased virulence. **(C)** With the assistance of Hfq, sRNA Qrr can base pair with the leader sequence of the DGC gene *vca0939* mRNA to expose the ribosome binding site for increasing Vca0939 production, leading to increasing c-di-GMP level and biofilm formation.

**FIGURE 4:**
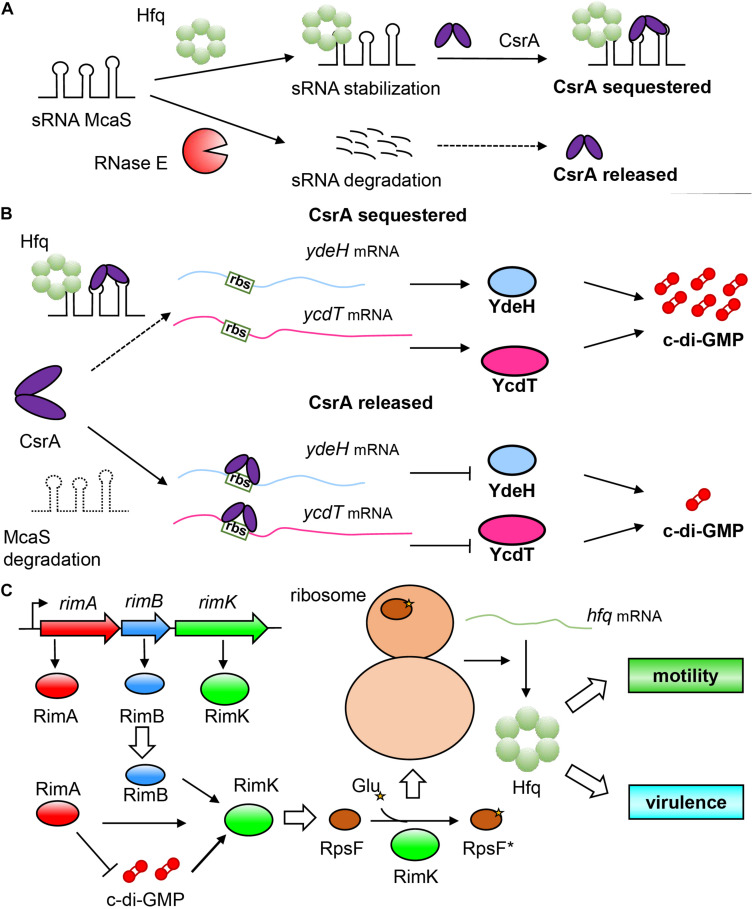
Indirect and mutual regulation between Hfq and c-di-GMP systems. **(A)** A complicated regulatory relationship between sRNA McaS and RNA binding proteins Hfq and CsrA. sRNA McaS can bind to two RNA binding proteins CsrA and Hfq through different binding motifs. When Hfq is present, McaS is stabilized to adopt a conformation non-degradable by RNase E. The stable McaS then binds with CsrA, disabling its regulation on target mRNA; on the contrary, when Hfq is not present, McaS will be degraded, allowing free CsrA to play a regulatory role on target mRNA. **(B)** Hfq regulates the concentration of c-di-GMP by affecting the stability of sRNA McaS, thereby causing McaS to capture CsrA. In the presence of Hfq, McaS adopts a stable conformation, and binds strongly with CsrA to prevent it from blocking the ribosome binding sites of *ydeH* and *ycdT* mRNA, so that they can be translated normally (The above is reasonable speculation); while without Hfq, McaS cannot adopt a stable conformation to capture CsrA, which is then released to bind to the ribosome binding site of *ydeH* and *ycdT* mRNA, resulting in translation inhibition. **(C)** c-di-GMP and RimA, RimB, RimK, and RpsF co-regulate regulate Hfq. RimA (a PDE), RimB and RimK are encoded by the operon rimABK. Importantly, RimK is a glutamate ligase, which can transfer glutamate residues to the C-terminus of RpsF (Protein S6), so that RpsF is activated to become RpsF* (asterisks indicate the glutamate residues), which can interact with ribosomal proteins to regulate downstream gene activity and fine-tune the entire proteome of bacteria, thereby affecting the expression of Hfq protein.

The type of RNA most regulated by Hfq is called sRNA, and Hfq participates in the post-transcriptional regulation of many genes via it, which is a type of non-coding RNA with a length between 37 and 500 nt, and can control the translation of target mRNA through many different mechanisms. Yet, in some regulatory pathways, Hfq can also directly regulate the translation of some mRNAs without the involvement of sRNA (Ellis et al., 2015; Chen and Gottesman, 2017). Hfq regulates RNA function mainly through the following six mechanisms: (1) Hfq refolds and forms stable complexes with certain sRNA, making it difficult to be degraded by relevant nucleases (Sledjeski et al., 2001; Møller et al., 2002; Massé et al., 2003) (Figure 1A); (2) Hfq binds and rearranges the leader sequence of mRNA to form a stem-loop structure within the ribosome binding site (rbs), which can block the binding of ribosome, and causes the target mRNA to be rapidly degraded by RNase E (Vytvytska et al., 1998, 2000; Vecerek et al., 2005; Salvail et al., 2013; Sonnleitner and Bläsi, 2014; Ellis et al., 2015; Chen and Gottesman, 2017; Figure 1B); (3) Hfq binds to mRNA, enabling polyA polymerase I (PAP I) to easily carry out polyadenylation of mRNA, thereby promoting the degradation of mRNA by exoribonuclease (Exo) (Hajnsdorf and Régnier, 2000; Le Derout et al., 2003; Figure 1C); (4) Hfq combines with sRNA and mRNA to form a partial duplex, causing them to be simultaneously degraded by endonuclease RNaseE (Massé et al., 2003; Morita et al., 2005; Pfeiffer et al., 2009; Figure 1D); (5) Hfq captures the partially paired sRNA and mRNA of the leader sequence ribosome binding site to block the binding of ribosome and inhibit the downstream translation process by *cis*-repression (Figure 1E; Desnoyers and Massé, 2012; Chen and Gottesman, 2017); (6) Hfq binds sRNA and mRNA to expose the mRNA ribosome binding site, thereby initiating translation process by *trans*-activation (Sittka et al., 2008; Figure 1F). The multiple regulation modes of RNA function triggered by Hfq means that Hfq is a rather flexible RNA matchmaker in bacteria (Møller et al., 2002; Zhang et al., 2002; Hämmerle et al., 2012; Updegrove et al., 2016).

Hfq can bind to many sRNAs and mRNAs (Małecka and Woodson, 2021), which are called Hfq-dependent RNAs (Kavita et al., 2018). With the help of Hfq, sRNA and/or mRNA become feasible to perform their functions and regulate various bacterial physiology by forming a Hfq-dependent regulatory network. Hfq is thus considered a genuine global post-transcriptional regulator in bacteria (Kavita et al., 2018), and the loss of Hfq has been found to cause substantial changes in the physiological phenotypes of many key bacteria, such as bacterial growth in *Yersinia enterocolitica* (Kakoschke et al., 2014), biofilm formation in *Xanthomonas axonpodis* pv. *citri.* (Liu et al., 2019), regulation of carbon catabolite repression (CCR) in *Pseudomonas aeruginosa* (Sonnleitner et al., 2018), quorum sensing in *Vibrio harveyi* and *Vibrio cholerae* (Lenz et al., 2004), and virulence of the pathogen *Haemophilus ducreyi*, etc., (Gangaiah et al., 2014; Feliciano et al., 2016; Kakoschke et al., 2016).

In addition, it was also found that Hfq acts as a new ribosome biogenesis factor by binding rRNA to affect rRNA processing, ribosome assembly, translational efficiency, and translational fidelity (Andrade et al., 2018; Sharma et al., 2018; Cai et al., 2019); or adjust the accuracy of protein synthesis by linking with tRNA (Lee and Feig, 2008). Meanwhile, Hfq can also bind to DNA to play other regulatory roles in certain DNA metabolism, such as DNA compaction and DNA replication (Updegrove et al., 2010; Jiang et al., 2015; Cech et al., 2016; Malabirade et al., 2017). Moreover, Hfq can combine with various enzymes such as RNase E, polynucleotide phosphorylase (PNPase), and PAP I to regulate their activities through protein-protein interactions (Mohanty et al., 2004; Morita et al., 2005). Further, Hfq-dependent regulation can also occur at the transcriptional level by affecting other unknown intermediate factors (Bellows et al., 2012; Dos Santos et al., 2019).

## Cyclic di-GMP (c-di-GMP)

C-di-GMP is currently the widely concerned and studied nucleotide second messenger molecule that plays a central role in regulating bacterial metabolism. It is synthesized from two molecules of GTP by diguanylate cyclase (DGC) containing a GG(D/E)EF domain (Valentini and Filloux, 2019), and degraded into pGpG or GMP by the c-di-GMP-specific phosphodiesterase (PDE) containing an EAL domain or HD-GYP domain, respectively (Tang et al., 2016; Zhou et al., 2016; Fu et al., 2018; Figure 2). The activities of these c-di-GMP metabolic enzymes are regulated by specific extracellular or intracellular signals, which directly affect the intracellular level of c-di-GMP. Changes in the intracellular c-di-GMP concentration can then affect bacterial motility (Wang et al., 2018), cell cycle (Kaczmarczyk et al., 2020), differentiation and morphology (Povolotsky and Hengge, 2012), biofilm formation, extracellular polysaccharides (Ha and O’Toole, 2015; Schmid et al., 2018), synthesis and degradation of adhesion factors (Hu et al., 2013; Floyk et al., 2020), expression of pathogenic virulence factors (Cotter and Stibitz, 2007), and production of antibiotics, such as HSAF (Heat Stable Antifungal Factor) (Xu et al., 2018, 2021; Han et al., 2020), as well as the host’s immune system (Burdette et al., 2011; Kobayashi et al., 2015) through binding to appropriate receptors.

The reason why c-di-GMP can regulate so many different physiological functions is because bacteria contain a wide variety of receptors (Chou and Galperin, 2016). The c-di-GMP receptors discovered to date include mainly the following categories: (1) proteins involved in transcriptional control (Li et al., 2018); (2) proteins containing a PilZ domain in pilus regulation, such as YcgR, which is the first known type of c-di-GMP receptors (Amikam and Galperin, 2006; Chin et al., 2012); (3) proteins containing degenerate GG(D/E)EF and/or EAL domains (Duerig et al., 2009); (4) proteins containing the high-affinity c-di-GMP-binding MshEN domain (Wang et al., 2016); (5) proteins involved in RNA degradation such as PNPase (Tuckerman et al., 2011); (6) proteins containing the GIL domain (GGDEF I-site like domain). The binding of such proteins to c-di-GMP depends on the RxGD motif in the GIL domain, which is comparable to the I-site in GGDEF domain (Fang et al., 2014); (7) trigger PDEs, which are multifunctional and can directly and specifically interact with macromolecular targets to regulate their activities through the binding and degradation of c-di-GMP, such as *E. coli* PdeR and PdeL (Hengge, 2016); and (8) RNA riboswitches (Smith and Strobel, 2011; Figure 2).

## Multiple Regulatory Relationship Between Hfq and c-di-GMP

Hfq and c-di-GMP can each play a pivotal regulatory role in the environmental adaptation of bacteria, but it is more interesting to find that both Hfq and c-di-GMP affect some common physiological activities. We will summarize existing reports and explain in detail the current understanding of regulatory relationship between them.

### Similarities Between Hfq and c-di-GMP in Regulating the Physiological Phenotypes of Bacteria

It is well known that both Hfq and c-di-GMP have their own unique functions and are involved in regulating different physiological activities, for example, Hfq can regulate DNA transaction independently (Cech et al., 2016), while c-di-GMP can affect chemotaxis (Suchanek et al., 2020) in *E. coli*. Meanwhile, both Hfq and c-di-GMP have also been found to have similar abilities in regulating various bacterial phenotypes such as the bacterial motility, biofilm formation, and virulence. In bacterial motility, Hfq regulates the expression level of flagellar synthesis regulation gene *flhDC* (co-transcribed from a promoter) by regulating five sRNAs to influence the motility of *E. coli* (De Lay and Gottesman, 2012). In contrast, c-di-GMP binds to YcgR (a flagellar brake protein), thereby affecting the interactions between YcgR and flagellar motor proteins MotA, FliG, and FliM to affect the motility of *E. coli*. (Nieto et al., 2019; Hou et al., 2020). In this way, both Hfq and c-di-GMP can regulate bacterial motility through different regulatory pathways, but whether there is regulatory relationship between the two systems remain unknown.

In the formation of bacterial biofilm, researchers have also found that both Hfq and c-di-GMP play similar regulatory functions in some bacteria. For example, in the human infection bacterium *Acinetobacter baumannii*, researchers found that deletion of Hfq significantly reduces its biofilm formation and infection (Kuo et al., 2017). At the same time, when the expression of c-di-GMP-specific metabolic enzyme (such as DGC A1S_3296) is changed, it will cause fluctuation of the intracellular c-di-GMP concentration, thereby also affecting bacterial biofilm formation (Ahmad et al., 2020). It is unclear how Hfq or c-di-GMP regulates the biofilm formation in *A*. *baumannii*.

Researchers also found that Hfq and c-di-GMP are both involved in regulating the virulence of certain pathogenic bacteria. For example, the *hfq* knockout strain in the opportunistic pathogen *P*. *aeruginosa* showed significantly reduced virulence in mice (Sonnleitner et al., 2003), while Kulasakara et al. (2006) also found that c-di-GMP metabolic enzymes that directly affect c-di-GMP concentration also regulate the virulence of *P*. *aeruginosa*. Subsequently, many researchers have started to explore the mechanisms by which Hfq and c-di-GMP co-regulate the virulence of *P*. *aeruginosa* (Wei et al., 2019; Janssen et al., 2020). Recently, it was discovered that Vfr is a virulence transcription factor in *P*. *aeruginosa*, which is affected by Hfq and c -di-GMP (Almblad et al., 2015; Janssen et al., 2020). Further, Vfr binds with another second messenger cAMP to form a complex cAMP-Vfr signal pathway, which is used to regulate the virulence of *P*. *aeruginosa*. Hfq can indirectly regulate Vfr while c-di-GMP can reduce the amount of cAMP to inhibit the cAMP-Vfr signal pathway. These evidences prove that Hfq and c-di-GMP can jointly participate in the regulation of virulence of *P. aeruginosa*, but how do Hfq and c-di-GMP establish regulatory relationship requires more experiments.

In addition, in several Gram-negative bacteria such as *Xanthomonas* (Yang et al., 2015, 2019; Lai et al., 2018; Xue et al., 2018; Liu et al., 2019), *Vibrio* (Tischler and Camilli, 2004, 2005; Liu et al., 2011; Bardill and Hammer, 2012; Zamorano-Sánchez et al., 2019; Wu et al., 2020), and in certain Gram-positive bacteria such as *Bacillus* (Chen et al., 2012; Fagerlund et al., 2016; Jagtap et al., 2016; Fu et al., 2018), both Hfq and c-di-GMP are found to participate in the regulation of phenotypes such as motility, biofilm formation and virulence, which demonstrate well that both Hfq and c-di-GMP can co-regulate certain phenotype in different bacteria. We thus assume that there is a certain relationship between the Hfq regulatory system and c-di-GMP signal transduction system.

### Hfq Directly Regulates the Intracellular c-di-GMP Concentration

As early as 2012, it was found that Hfq directly regulates the activity of c-di-GMP metabolic enzymes and concentration of c-di-GMP in *Yersinia pestis* that is a pathogen of plague belonging to the category A infectious disease. It was discovered that the biofilm formation of *Y. pestis* can help it to colonize in flea vector and further spread from the flea intermediate to the final mammalian host (Hinnebusch and Erickson, 2008). Studies also showed that its biofilm formation is controlled by the synthesis of extracellular polysaccharides regulated by c-di-GMP (Bobrov et al., 2008, 2011). Bellows et al. (2012) found that *Y*. *pestis* Hfq promoted the expression of the PDE-encoding gene *hmsP* at the transcription level, and diminished the post-transcriptional level of the DGC-encoding gene *hmsT* by reducing the stability of its mRNA. The combination of the decrease of DGC and increase of PDE will cause a large decrease in intracellular c-di-GMP level, which will ultimately affect the formation of bacterial biofilm (Figure 3A). It is the first example that Hfq directly regulates amounts of the c-di-GMP metabolic enzymes, and it seems to occur at different transcriptional and post-transcriptional levels (Bellows et al., 2012).

Another example is for the soft rot pathogen *Dickeya dadantii*. Yuan et al. (2019) found that Hfq can simultaneously inhibit the production of two DGCs, namely, GcpA and GcpL, which significantly reduces the concentration of intracellular c-di-GMP and affects the motility and virulence (Figure 3B). In this way, Hfq directly regulates the expression of c-di-GMP metabolic enzymes and affects the concentration of intracellular c-di-GMP, which is the most direct way for regulatory relationship between the two factors. Hfq can up-regulate or down-regulate the same metabolic enzyme at the same time (Figure 3B), or up-regulates one DGC/PDE while down-regulates another PDE/DGC (Figure 3A) to achieve a synergistic effect by Hfq. However, the authors of these two research articles did not conduct further in-depth research on their mechanisms. Whether some sRNAs were involved in the regulation of c-di-GMP metabolic enzymes by Hfq is still a question worthy of exploring.

In *V*. *cholerae*, it has been reported that some sRNAs can participate in the regulation of c-di-GMP metabolic enzymes by Hfq (Zhao X. et al., 2013). For example, Qrr (quorum regulatory RNA) is a sRNA that has been proven to regulate quorum sensing (Lenz et al., 2004), and Vca0939 is a DGC in *V*. *cholerae* (Hammer and Bassler, 2007). Zhao X. et al. (2013) found that in *V*. *cholerae*, Qrr can base pair with the leader sequence of *vca0939* mRNA with the help of Hfq to expose the ribosome binding site and initiate the expression of *vca0939*, thereby causing increase in the intracellular c-di-GMP concentration and biofilm formation (Figure 3C).

The above evidence is the strongest proof for direct crosstalk between Hfq and c-di-GMP. We believe that through in-depth exploration, more and more cases of direct regulation between Hfq and c-di-GMP can be found. However, so far, there is no case where c-di-GMP can directly regulate the Hfq system.

### Indirect Regulation Between Hfq and c-di-GMP

Hfq can not only control the concentration of intracellular c-di-GMP directly by regulating the expression of c-di-GMP metabolic enzymes, but also exhibits regulation with c-di-GMP signal transduction systems indirectly through other regulatory factors.

A representative example of Hfq indirectly regulating c-di-GMP in *E. coli* is through sRNA, namely, the McaS system (Jørgensen et al., 2013). McaS is a dual-functional sRNA related to multicellular adhesion and can combine with Hfq or other RNA-binding protein CsrA by using its different motifs (Holmqvist and Vogel, 2013). Similarly, the regulatory mechanism of CsrA on mRNA also appears to be diverse (Romeo and Babitzke, 2018). It can not only inhibit the initiation of translation by blocking the binding of ribosomes to target mRNA, leading to instability of mRNA (Baker et al., 2002), but can also participate in the transcription attenuation and mediate the Rho-dependent termination of some mRNA (Figueroa-Bossi et al., 2014). Furthermore, it can also prevent the 5′-end-dependent cleavage by RNase E to stabilize mRNA (Yakhnin et al., 2013). McaS and the two powerful RNA-binding proteins (Hfq and CsrA) thus form a complex regulatory network as shown in Figure 4A. McaS relies on the binding with Hfq to enhance its stability and prevent cleavage and degradation by RNase E (Thomason et al., 2012; Jørgensen et al., 2013). The non-degradable McaS can then bind to CsrA through its loop GGA nucleotide sequence to sequester CsrA and prevent the downstream regulation by CsrA. In addition, it is worth mentioning that the downstream binding target of CsrA also contains *hfq* mRNA. CsrA can inhibit the translation of Hfq by binding to the site overlapping with its Shine-Dalgarno (SD) sequence (Baker et al., 2007). Moreover, CsrA can even titrate sRNAs away from Hfq to interfere with their ability to perform Hfq-dependent regulation (Kavita et al., 2018), which further enhances the complexity of the regulatory network. It is due to the regulatory relationship between the three factors that Hfq realizes the indirect regulation of the intracellular c-di-GMP concentration in bacteria. The specific regulatory mechanism we speculate is as follows: When Hfq is present, it can bind to McaS to enhance its stability, which can tightly bind CsrA, leaving no free CsrA (CsrA is sequestered in this case) to block the ribosome binding sites of *ydeH* and *ycdT* mRNAs, resulting in successful translation of these two DGC genes, and increasing intracellular c-di-GMP concentration. However, in the absence of Hfq, McaS is unstable and cannot bind with CsrA (Holmqvist et al., 2016), which can then bind to the ribosome binding sites of *ydeH* and *ycdT* mRNA, thereby inhibits the translation of *ydeH* and *ycdT*; this event leads to a decrease in intracellular c-di-GMP concentration, and results in enhanced bacterial motility and reduced biofilm formation (Jørgensen et al., 2013; Figure 4B). In this model, Hfq does not directly participate in the regulation of c-di-GMP level, but affects the stability of McaS to influence the binding between McaS and CsrA and indirectly affect the intracellular c-di-GMP concentration, which in turn affects the phenotypic changes such as motility and biofilm formation. The discovery of multifunctional sRNAs such as McaS not only enriches the physiological activities of sRNA regulation in bacteria (Leistra et al., 2019), but also reflects the complexity of relationship between Hfq and c-di-GMP.

Other than these data, there are also many reports indicating that Hfq can regulate the intracellular c-di-GMP concentration through unknown mechanisms. For example, Pusic et al. (2018) found that in *P*. *aeruginosa*, when *hfq* is deleted, the bacterial biofilm formation is significantly reduced with a large decrease in intracellular c-di-GMP concentration, but analysis of the RNA-seq of *hfq* deletion mutant showed that the transcriptional level of c-di-GMP metabolic enzyme-relevant genes did not change significantly. Therefore, its true regulatory mechanism remains to be further explored. In many bacteria, Hfq may be able to indirectly regulate c-di-GMP metabolic enzymes at the transcriptional (like HmsP) (Bellows et al., 2012), post-transcriptional, translational, and post-translational levels, or even through protein-protein interaction, signal stimulation, and so on.

When Corona et al. (2018) studied the function of catabolite repression control protein Crc involved in CCR in *P*. *aeruginosa*, they found that Hfq can establish an indirect relationship with c-di-GMP. Using the post-transcriptional regulation variation (PTV) parameters, they combined the analysis of transcriptome and proteome to extract information at the post-transcriptional level, confirming that Crc exhibits a role in influencing the c-di-GMP signal transduction system. Sonnleitner et al. (2018) reported that Crc, Hfq, and CrcZ sRNA cooperate to regulate CCR, and Crc is perhaps involved in the regulation of the c-di-GMP signal transduction system through transcriptome analyses. We therefore speculate that Hfq and c-di-GMP are jointly involved in the regulation of CCR, but it is still unknown whether the indirect regulation between the two is established through Crc.

Conversely, c-di-GMP can also control Hfq by fine-tuning intermediate factors (Figure 4C). In *Pseudomonas fluorescens*, the operon *rimABK* encodes three proteins: RimA (a PDE), RimB, and RimK. RimB is currently a hypothetical protein with unknown function, mainly playing a supporting role in the function of *rimABK* operon (Little et al., 2016). RimK is, intriguingly, an ATP-dependent glutamate ligase (Kang et al., 1989) that can append glutamate residues to the C-terminus of protein S6 (RpsF) located in the ribosomal 30S subunit (Zhao G. et al., 2013). By regulating the function of the posttranslational modification of ribosomal protein RpsF, RimK can control the translation of some relevant proteins (Kino et al., 2011; Grenga et al., 2020). Studies have shown that RimA, RimB and c-di-GMP can all activate RimK (Figure 4C). It is worth noting that RimA plays a very crucial role in this system, which is very similar to the “trigger PDE” as mentioned above, and can not only interact directly with RimK to moderately stimulate its activity, but also degrade c-di-GMP to negatively regulate RimK. In this way, bacteria can “fine-tune” their proteome through this mechanism to respond appropriately to the surrounding environment (Little et al., 2016). Therefore, c-di-GMP can indirectly regulate Hfq through the intermediate factor of RimK.

The indirect regulation between Hfq and c-di-GMP may be carried out through different intermediate factors in different bacteria, which is related to the characteristics of the bacteria themselves, indicating that the indirect regulation between Hfq and c-di-GMP is worth conducting with in-depth research. But, can Hfq and c-di-GMP establish an indirect mutual regulation relationship through other means? Some scholars have mentioned a concept of “GTP-pool” (Lopez, 1982). Indeed, GTP in the cell is a substrate involved in a variety of biological metabolic pathways and is very important for bacterial growth and feedback of environmental signals. In addition, c-di-GMP is synthesized by two molecules of GTP. Therefore, when the GTP-pool is depleted (Ölschläger et al., 2011), it may directly affect the synthesis of c-di-GMP. As an important global transcriptional regulator in bacteria, Hfq may exhibit a greater impact if it is deleted. Whether it will affect the GTP-pool and cause the depletion of the GTP-pool to regulate the synthesis of c-di-GMP is our future focus.

## Conclusion

Although the current understanding on the multiple regulatory relationship between Hfq regulatory and c-di-GMP signal transduction systems remains to be further explored, it is clear at the moment that: (1) Hfq can directly regulate c-di-GMP metabolic enzymes at the post-transcriptional and translational levels, thereby regulating the intracellular c-di-GMP level and affecting the physiological functions of bacteria; (2) Hfq can indirectly regulate genes encoding c-di-GMP metabolic enzyme through other intermediate factors, and establishes an indirect regulation with c-di-GMP; (3) c-di-GMP may indirectly regulate Hfq through certain intermediate factors, but this conclusion needs to be further verified. In a word, the above-mentioned multiple regulatory relationship between Hfq and c-di-GMP have been uncovered somewhat, but requires further and deeper exploration.

The RNA chaperone protein Hfq is an extremely important post-transcriptional regulatory factor in bacteria, and the second messenger c-di-GMP is also a key factor in the bacterial signal transduction system. This review elaborated on the currently reported evidence of direct and indirect regulation between Hfq and c-di-GMP, which not only links the two regulatory systems together, but also establishes possible connections with other regulatory systems to lay a more solid foundation for bacterial signaling network. We believe that in the future more complex physiological metabolic systems will be greatly studied and a more complete global regulatory network in bacteria can be drawn. In particular, comprehensive exploration of the multiple regulatory relationship between the two in certain highly infectious pathogens will provide new research ideas for their pathogenic mechanisms and their complex interactions between pathogens and hosts, thereby providing better strategies for the prevention and treatment of pathogenic bacteria.

## Author Contributions

YF and ZY wrote the original draft. JH and S-HC raised issues and revised the manuscript. LZ and ZL designed and drew figures. WY and XS sorted out the reference materials. QT and JH coordinated the work and secured research funding. All authors contributed to the article and approved the submitted version.

## Conflict of Interest

The authors declare that the research was conducted in the absence of any commercial or financial relationships that could be construed as a potential conflict of interest.

## References

[B1] Ahmad I. Nygren E. Khalid F. Myint S. L. Uhlin B. E. (2020). A Cyclic-di-GMP signalling network regulates biofilm formation and surface associated motility of *Acinetobacter baumannii* 17978. *Sci. Rep.* 10:1991. 10.1038/s41598-020-58522-5 32029764 PMC7005169

[B2] Almblad H. Harrison J. J. Rybtke M. Groizeleau J. Givskov M. Parsek M. R. (2015). The cyclic AMP-Vfr signaling pathway in *Pseudomonas aeruginosa* is inhibited by cyclic di-GMP. *J. Bacteriol.* 197 2190–2200. 25897033 10.1128/JB.00193-15 PMC4455276

[B3] Amikam D. Galperin M. Y. (2006). PilZ domain is part of the bacterial c-di-GMP binding protein. *Bioinformatics* 22 3–6. 10.1093/bioinformatics/bti739 16249258

[B4] Andrade J. M. Dos Santos R. F. Chelysheva I. Ignatova Z. Arraiano C. M. (2018). The RNA-binding protein Hfq is important for ribosome biogenesis and affects translation fidelity. *EMBO J.* 37:e97631. 10.15252/embj.201797631 PMC5983149 29669858

[B5] Baker C. S. Eöry L. A. Yakhnin H. Mercante J. Romeo T. Babitzke P. (2007). CsrA inhibits translation initiation of *Escherichia coli* hfq by binding to a single site overlapping the Shine-Dalgarno sequence. *J. Bacteriol.* 189 5472–5481. 10.1128/JB.00529-07 17526692 PMC1951803

[B6] Baker C. S. Morozov I. Suzuki K. Romeo T. Babitzke P. (2002). CsrA regulates glycogen biosynthesis by preventing translation of glgC in *Escherichia coli*. *Mol. Microbiol.* 44 1599–1610. 10.1046/j.1365-2958.2002.02982.x 12067347

[B7] Bardill J. P. Hammer B. K. (2012). Non-coding sRNAs regulate virulence in the bacterial pathogen *Vibrio cholerae*. *RNA Biol.* 9 392–401. 10.4161/rna.19975 22546941 PMC3384565

[B8] Beich-Frandsen M. Večerek B. Sjöblom B. Bläsi U. Djinović-Carugo K. (2011). Structural analysis of full-length Hfq from *Escherichia coli*. *Acta Crystallogr. Sect. F Struct. Biol. Cryst. Commun.* 67 536–540. 10.1107/S174430911100786X 21543856 PMC3087635

[B9] Bellows L. E. Koestler B. J. Karaba S. M. Waters C. M. Lathem W. W. (2012). Hfq-dependent, co-ordinate control of cyclic diguanylate synthesis and catabolism in the plague pathogen *Yersinia pestis*. *Mol. Microbiol.* 86 661–674. 10.1111/mmi.12011 22924957 PMC3480973

[B10] Bobrov A. G. Kirillina O. Forman S. Mack D. Perry R. D. (2008). Insights into *Yersinia pestis* biofilm development: topology and co-interaction of Hms inner membrane proteins involved in exopolysaccharide production. *Environ. Microbiol.* 10 1419–1432. 10.1111/j.1462-2920.2007.01554.x 18279344

[B11] Bobrov A. G. Kirillina O. Ryjenkov D. A. Waters C. M. Price P. A. Fetherston J. D. (2011). Systematic analysis of cyclic di-GMP signalling enzymes and their role in biofilm formation and virulence in *Yersinia pestis*. *Mol. Microbiol.* 79 533–551. 10.1111/j.1365-2958.2010.07470.x 21219468 PMC3058942

[B12] Boyd C. D. O’Toole G. A. (2012). Second messenger regulation of biofilm formation: breakthroughs in understanding c-di-GMP effector systems. *Annu. Rev. Cell Dev. Biol.* 28 439–462. 10.1146/annurev-cellbio-101011-155705 23057745 PMC4936406

[B13] Burdette D. L. Monroe K. M. Sotelo-Troha K. Iwig J. S. Eckert B. Hyodo M. (2011). STING is a direct innate immune sensor of cyclic di-GMP. *Nature* 478 515–518. 21947006 10.1038/nature10429 PMC3203314

[B14] Cai Q. Wang G. Li Z. Zhang L. Fu Y. Yang X. (2019). SWATH based quantitative proteomics analysis reveals Hfq2 play an important role on pleiotropic physiological functions in *Aeromonas hydrophila*. *J. Proteomics* 195 1–10. 10.1016/j.jprot.2018.12.030 30597314

[B15] Cech G. M. Szalewska-Pałasz A. Kubiak K. Malabirade A. Grange W. Arluison V. (2016). The *Escherichia coli* Hfq protein: an unattended DNA-transactions regulator. *Front. Mol. Biosci.* 3:36. 10.3389/fmolb.2016.00036 27517037 PMC4963395

[B16] Chen J. Gottesman S. (2017). Hfq links translation repression to stress-induced mutagenesis in E. coli. *Genes Dev.* 31 1382–1395. 10.1101/gad.302547.117 28794186 PMC5580658

[B17] Chen Y. Chai Y. Guo J. H. Losick R. (2012). Evidence for cyclic di-GMP-mediated signaling in *Bacillus subtilis*. *J. Bacteriol.* 194 5080–5090. 10.1128/JB.01092-12 22821967 PMC3430322

[B18] Chin K. H. Kuo W. T. Yu Y. J. Liao Y. T. Yang M. T. Chou S. H. (2012). Structural polymorphism of c-di-GMP bound to an EAL domain and in complex with a type II PilZ-domain protein. *Acta Crystallogr. D Biol. Crystallogr.* 68 1380–1392. 10.1107/S0907444912030594 22993092

[B19] Chou S. H. Galperin M. Y. (2016). Diversity of cyclic Di-GMP-binding proteins and mechanisms. *J. Bacteriol.* 198 32–46. 26055114 10.1128/JB.00333-15 PMC4686193

[B20] Corona F. Reales-Calderón J. A. Gil C. Martínez J. L. (2018). The development of a new parameter for tracking post-transcriptional regulation allows the detailed map of the *Pseudomonas aeruginosa* Crc regulon. *Sci. Rep.* 8:16793. 10.1038/s41598-018-34741-9 30429516 PMC6235884

[B21] Cotter P. A. Stibitz S. (2007). c-di-GMP-mediated regulation of virulence and biofilm formation. *Curr. Opin. Microbiol.* 10 17–23. 17208514 10.1016/j.mib.2006.12.006

[B22] De Lay N. Gottesman S. (2012). A complex network of small non-coding RNAs regulate motility in *Escherichia coli*. *Mol. Microbiol.* 86 524–538. 10.1111/j.1365-2958.2012.08209.x 22925049 PMC7458410

[B23] Desnoyers G. Massé E. (2012). Noncanonical repression of translation initiation through small RNA recruitment of the RNA chaperone Hfq. *Genes Dev.* 26 726–739. 22474262 10.1101/gad.182493.111 PMC3323883

[B24] Dos Santos R. F. Arraiano C. M. Andrade J. M. (2019). New molecular interactions broaden the functions of the RNA chaperone Hfq. *Curr. Genet.* 65 1313–1319. 10.1007/s00294-019-00990-y 31104083

[B25] Duerig A. Abel S. Folcher M. Nicollier M. Schwede T. Amiot N. (2009). Second messenger-mediated spatiotemporal control of protein degradation regulates bacterial cell cycle progression. *Genes Dev.* 23 93–104. 10.1101/gad.502409 19136627 PMC2632171

[B26] Ellis M. J. Trussler R. S. Haniford D. B. (2015). Hfq binds directly to the ribosome-binding site of IS10 transposase mRNA to inhibit translation. *Mol. Microbiol.* 96 633–650. 10.1111/mmi.12961 25649688 PMC5006887

[B27] Fagerlund A. Smith V. Røhr ÅK. Lindbäck T. Parmer M. P. Andersson K. K. (2016). Cyclic diguanylate regulation of *Bacillus cereus* group biofilm formation. *Mol. Microbiol.* 101 471–494. 10.1111/mmi.13405 27116468

[B28] Fang X. Ahmad I. Blanka A. Schottkowski M. Cimdins A. Galperin M. Y. (2014). GIL, a new c-di-GMP-binding protein domain involved in regulation of cellulose synthesis in enterobacteria. *Mol. Microbiol.* 93 439–452. 10.1111/mmi.12672 24942809 PMC4116459

[B29] Feliciano J. R. Grilo A. M. Guerreiro S. I. Sousa S. A. Leitão J. H. (2016). Hfq: a multifaceted RNA chaperone involved in virulence. *Future Microbiol.* 11 137–151. 26685037 10.2217/fmb.15.128

[B30] Figueroa-Bossi N. Schwartz A. Guillemardet B. D’Heygčre F. Bossi L. Boudvillain M. (2014). RNA remodeling by bacterial global regulator CsrA promotes Rho-dependent transcription termination. *Genes Dev.* 28 1239–1251. 10.1101/gad.240192.114 24888591 PMC4052769

[B31] Floyk K. A. Lee C. K. Xian W. Nametalla M. Valentine A. Crair B. (2020). c-di-GMP modulates type IV MSHA pilus retraction and surface attachment in *Vibrio cholerae*. *Nat. Commun.* 11:1549. 10.1038/s41467-020-15331-8 32214098 PMC7096442

[B32] Franze de Fernandez M. T. Eoyang L. August J. T. (1968). Factor fraction required for the synthesis of bacteriophage Qβ-RNA. *Nature* 219 588–590. 10.1038/219588a0 4874917

[B33] Fu Y. Yu Z. Liu S. Chen B. Zhu L. Li Z. (2018). c-di-GMP regulates various phenotypes and insecticidal activity of gram-positive *Bacillus thuringiensis*. *Front. Microbiol.* 9:45. 10.3389/fmicb.2018.00045 29487570 PMC5816809

[B34] Gangaiah D. Labandeira-Rey M. Zhang X. Fortney K. R. Ellinger S. Zwickl B. (2014). *Haemophilus ducreyi* Hfq contributes to virulence gene regulation as cells enter stationary phase. *mBio* 5:e001081-13. 10.1128/mBio.01081-13 24520065 PMC3950518

[B35] Grenga L. Little R. H. Chandra G. Woodcock S. D. Saalbach G. Morris R. J. (2020). Control of mRNA translation by dynamic ribosome modification. *PLoS Genet.* 16:e1008837. 10.1371/journal.pgen.1008837 PMC7343187 32584816

[B36] Ha D. G. O’Toole G. A. (2015). c-di-GMP and its effects on biofilm formation and dispersion: a *Pseudomonas aeruginosa* review. *Microbiol. Spectr.* 3:MB-0003-2014. 10.1128/microbiolspec.MB-0003-2014 26104694 PMC4498269

[B37] Hajnsdorf E. Régnier P. (2000). Host factor Hfq of *Escherichia coli* stimulates elongation of poly(A) tails by poly(A) polymerase I. *Proc. Natl. Acad. Sci. U. S. A.* 97 1501–1505. 10.1073/pnas.040549897 10677490 PMC26463

[B38] Hammer B. K. Bassler B. L. (2007). Regulatory small RNAs circumvent the conventional quorum sensing pathway in pandemic *Vibrio cholerae*. *Proc. Natl. Acad. Sci. U. S. A.* 104 11145–11149. 10.1073/pnas.0703860104 17556542 PMC1888797

[B39] Hämmerle H. Beich-Frandsen M. Večerek B. Rajkowitsch L. Carugo O. Djinović-Carugo K. (2012). Structural and biochemical studies on ATP binding and hydrolysis by the *Escherichia coli* RNA chaperone Hfq. *PLoS one* 7:e50892. 10.1371/journal.pone.0050892 23226421 PMC3511402

[B40] Han S. Shen D. Wang Y. C. Chou S. H. Gomelsky M. Gao Y. G. (2020). A YajQ-LysR-like, cyclic di-GMP-dependent system regulating biosynthesis of an antifungal antibiotic in a crop-protecting bacterium, Lysobacter enzymegenes. *Mol. Plant Pathol.* 21 218–229. 10.1111/mpp.12890 31747123 PMC6988422

[B41] Hengge R. (2016). Trigger phosphodiesterases as a novel class of c-di-GMP effector proteins. *Philos. Trans. R. Soc. Lond. B Biol. Sci.* 371:20150498. 10.1098/rstb.2015.0498 27672149 PMC5052742

[B42] Hengge R. (2021). High-specificity local and global c-di-GMP signaling. *Trends Microbiol.* 10.1016/j.tim.2021.02.003 Online ahead of print. 33640237

[B43] Hinnebusch B. J. Erickson D. L. (2008). *Yersinia pestis* biofilm in the flea vector and its role in the transmission of plague. *Curr. Top. Microbiol. Immunol.* 322 229–248. 10.1007/978-3-540-75418-3_11 18453279 PMC3727414

[B44] Holmqvist E. Vogel J. (2013). A small RNA serving both the Hfq and CsrA regulons. *Genes Dev.* 27 1073–1078. 10.1101/gad.220178.113 23699406 PMC3672642

[B45] Holmqvist E. Wright P. R. Li L. Bischler T. Barquist L. Reinhardt R. (2016). Global RNA recognition patterns of post-transcriptional regulators Hfq and CsrA revealed by UV crosslinking in vivo. *EMBO J.* 35 991–1011. 10.15252/embj.201593360 27044921 PMC5207318

[B46] Hou Y. J. Yang W. S. Hong Y. Zhang Y. Wang D. C. Li D. F. (2020). Structural insights into the mechanism of c-di-GMP-bound YcgR regulating flagellar motility in *Escherichia coli*. *J. Biol. Chem.* 295 808–821. 10.1074/jbc.RA119.009739 31836667 PMC6970932

[B47] Hu J. Wang B. Fang X. Means W. J. McCormick R. J. Gomelsky M. (2013). c-di-GMP signaling regulates E. coli O157:H7 adhesion to colonic epithelium. *Vet. Microbiol.* 164 344–351. 10.1016/j.vetmic.2013.02.023 23528649

[B48] Ishikawa H. Otaka H. Maki K. Morita T. Aiba H. (2012). The functional Hfq-binding module of bacterial sRNAs consists of a double or single hairpin preceded by a U-rich sequence and followed by a 3′ poly(U) tail. *RNA* 18 1062–1074. 10.1261/rna.031575.111 22454537 PMC3334693

[B49] Jagtap C. B. Kumar P. Rao K. K. (2016). *Bacillus subtilis* Hfq: a role in chemotaxis and motility. *J. Biosci.* 41 347–358. 10.1007/s12038-016-9618-9 27581927

[B50] Janssen K. H. Corley J. M. Djapgne L. Cribbs J. T. Voelker D. Slusher Z. (2020). Hfq and sRNA 179 inhibit expression of the *Pseudomonas aeruginosa* cAMP-Vfr and Type III secretion regulons. *mBio* 11:e00363-20. 10.1128/mBio.00363-20 32546612 PMC7298702

[B51] Jenal U. Malone J. (2006). Mechanisms of cyclic-di-GMP signaling in bacteria. *Annu. Rev. Genet.* 40 385–407. 16895465 10.1146/annurev.genet.40.110405.090423

[B52] Jiang K. Zhang C. Guttula D. Liu F. van Kan J. A. Lavelle C. (2015). Effects of Hfq on the conformation and compaction of DNA. *Nucleic Acids Res.* 43 4332–4341. 25824948 10.1093/nar/gkv268 PMC4417175

[B53] Jørgensen M. G. Thomason M. K. Havelund J. Valentin-Hansen P. Storz G. (2013). Dual function of the McaS small RNA in controlling biofilm formation. *Genes Dev.* 27 1132–1145. 10.1101/gad.214734.113 23666921 PMC3672647

[B54] Kaczmarczyk A. Hempel A. M. von Arx C. Böhm R. Dubey B. N. Nesper J. (2020). Precise timing of transcription by c-di-GMP coordinates cell cycle and morphogenesis in Caulobacter. *Nat. Commun.* 11:816. 10.1038/s41467-020-14585-6 32041947 PMC7010744

[B55] Kakoschke T. K. Kakoschke S. C. Zeuzem C. Bouabe H. Adler K. Heesemann J. (2016). The RNA chaperone Hfq is essential for virulence and modulates the expression of four adhesins in *Yersinia enterocolitica*. *Sci. Rep.* 6:29275. 10.1038/srep29275 27387855 PMC4937351

[B56] Kakoschke T. Kakoschke S. Magistro G. Schubert S. Borath M. Heesemann J. (2014). The RNA chaperone Hfq impacts growth, metabolism and production of virulence factors in *Yersinia enterocolitica*. *PLoS One* 9:e86113. 10.1371/journal.pone.0086113 24454955 PMC3893282

[B57] Kang W. K. Icho T. Isono S. Kitakawa M. Isono K. (1989). Characterization of the gene rimK responsible for the addition of glutamic acid residues to the C-terminus of ribosomal protein S6 in *Escherichia coli* K12. *Mol. Gen. Genet.* 217 281–288. 10.1007/BF02464894 2570347

[B58] Kavita K. de Mets F. Gottesman S. (2018). New aspects of RNA-based regulation by Hfq and its partner sRNAs. *Curr. Opin. Microbiol.* 42 53–61. 10.1016/j.mib.2017.10.014 29125938 PMC10367044

[B59] Kino K. Arai T. Arimura Y. (2011). Poly-α-glutamic acid synthesis using a novel catalytic activity of RimK from *Escherichia coli* K-12. *Appl. Environ. Microbiol.* 77 2019–2025. 10.1128/AEM.02043-10 21278279 PMC3067337

[B60] Kobayashi H. Kobayashi C. I. Nakamura-Ishizu A. Karigane D. Haeno H. Yamamoto K. N. (2015). Bacterial c-di-GMP affects hematopoietic stem/progenitors and their niches through STING. *Cell Rep.* 11 71–84. 10.1016/j.celrep.2015.02.066 25843711

[B61] Kulasakara H. Lee V. Brencic A. Liberati N. Urbach J. Miyata S. (2006). Analysis of *Pseudomonas aeruginosa* diguanylate cyclases and phosphodiesterases reveals a role for bis-(3′-5′)-cyclic-GMP in virulence. *Proc. Natl. Acad. Sci. U. S. A.* 103 2839–2844. 10.1073/pnas.0511090103 16477007 PMC1413825

[B62] Kuo H. Y. Chao H. H. Liao P. C. Hsu L. Chang K. C. Tung C. H. (2017). Functional characterization of *Acinetobacter baumannii* lacking the RNA chaperone Hfq. *Front. Microbiol.* 8:2068. 10.3389/fmicb.2017.02068 29163381 PMC5663733

[B63] Lai J. L. Tang D. J. Liang Y. W. Zhang R. Chen Q. Qin Z. P. (2018). The RNA chaperone Hfq is important for the virulence, motility and stress tolerance in the phytopathogen Xanthomonas campestris. *Environ. Microbiol. Rep.* 10 542–554. 10.1111/1758-2229.12657 29901272

[B64] Le Derout J. Folichon M. Briani F. Dehò G. Régnier P. Hajnsdorf E. (2003). Hfq affects the length and the frequency of short oligo(A) tails at the 3′ end of *Escherichia coli* rpsO mRNAs. *Nucleic Acids Res.* 31 4017–4023. 10.1093/nar/gkg456 12853618 PMC165971

[B65] Lee T. Feig A. L. (2008). The RNA binding protein Hfq interacts specifically with tRNAs. *RNA* 14 514–523. 18230766 10.1261/rna.531408 PMC2248270

[B66] Leistra A. N. Curtis N. C. Contreras L. M. (2019). Regulatory non-coding sRNAs in bacterial metabolic pathway engineering. *Metab. Eng.* 52 190–214. 10.1016/j.ymben.2018.11.013 30513348

[B67] Lenz D. H. Mok K. C. Lilley B. N. Kulkarni R. V. Wingreen N. S. Bassler B. L. (2004). The small RNA chaperone Hfq and multiple small RNAs control quorum sensing in *Vibrio harveyi* and *Vibrio cholerae*. *Cell* 118 69–82. 10.1016/j.cell.2004.06.009 15242645

[B68] Li W. Li M. Hu L. Zhu J. Xie Z. Chen J. (2018). HpoR, a novel c-di-GMP effective transcription factor, links the second messenger’s regulatory function to the mycobacterial antioxidant defense. *Nucleic Acids Res.* 46 3595–3611. 10.1093/nar/gky146 29490073 PMC5909442

[B69] Little R. H. Grenga L. Saalbach G. Howat A. M. Pfeilmeier S. Trampari E. (2016). Adaptive remodeling of the bacterial proteome by specific ribosomal modification regulates *Pseudomonas* infection and niche colonisation. *PLoS Genet.* 12:e1005837. 10.1371/journal.pgen.1005837 26845436 PMC4741518

[B70] Liu H. Wang Q. Liu Q. Cao X. Shi C. Zhang Y. (2011). Roles of Hfq in the stress adaptation and virulence in fish pathogen *Vibrio alginolyticus* and its potential application as a target for live attenuated vaccine. *Appl. Microbiol. Biotechnol.* 91 353–364. 10.1007/s00253-011-3286-3 21523476

[B71] Liu X. Yan Y. Wu H. Zhou C. Wang X. (2019). Biological and transcriptomic studies reveal hfq is required for swimming, biofilm formation and stress response in *Xanthomonas axonpodis* pv. citri. *BMC Microbiol.* 19:103. 10.1186/s12866-019-1476-9 31113370 PMC6530196

[B72] Lopez J. M. (1982). GTP pool expanision is necessary for the growth rate increase occurring in *Bacillus subtilis* after amino acids shift-up. *Arch. Microbiol.* 131 247–251. 10.1007/BF00405887 6808962

[B73] Malabirade A. Jiang K. Kubiak K. Diaz-Mendoza A. Liu F. van Kan J. A. (2017). Compaction and condensation of DNA mediated by the C-terminal domain of Hfq. *Nucleic Acids Res.* 45 7299–7308. 10.1093/nar/gkx431 28521053 PMC5499573

[B74] Małecka E. M. Woodson S. A. (2021). Stepwise sRNA targeting of structured bacterial mRNAs leads to abortive annealing. *Mol. Cell* 81 1988–1999. 33705712 10.1016/j.molcel.2021.02.019 PMC8106647

[B75] Massé E. Escorcia F. E. Gottesman S. (2003). Coupled degradation of a small regulatory RNA and its mRNA targets in *Escherichia coli*. *Genes Dev.* 17 2374–2383. 10.1101/gad.1127103 12975324 PMC218075

[B76] Mohanty B. K. Maples V. F. Kushner S. R. (2004). The Sm-like protein Hfq regulates polyadenylation dependent mRNA decay in *Escherichia coli*. *Mol. Microbiol.* 54 905–920. 10.1111/j.1365-2958.2004.04337.x 15522076

[B77] Møller T. Franch T. Højrup P. Keene D. R. Bächinger H. P. Brennan R. G. (2002). Hfq: a bacterial Sm-like protein that mediates RNA-RNA interaction. *Mol. Cell* 9 23–30. 11804583 10.1016/s1097-2765(01)00436-1

[B78] Morita T. Maki K. Aiba H. (2005). RNase E-based ribonucleoprotein complexes: mechanical basis of mRNA destabilization mediated by bacterial noncoding RNAs. *Genes Dev.* 19 2176–2186. 10.1101/gad.1330405 16166379 PMC1221888

[B79] Mura C. Randolph P. S. Patterson J. Cozen A. E. (2013). Archaeal and eukaryotic homologs of Hfq: a structural and evolutionary perspective on Sm function. *RNA Biol.* 10 636–651. 10.4161/rna.24538 23579284 PMC3710371

[B80] Nieto V. Partridge J. D. Severin G. B. Lai R. Z. Waters C. M. Parkinson J. S. (2019). Under elevated c-di-GMP in *Escherichia coli*, YcgR alters flagellar motor and speed sequentially, with additional negative control of the flagellar regulon via the adaptor protein RssB. *J. Bacteriol.* 202:e00578-19. 10.1128/JB.00578-19 31611290 PMC6932234

[B81] Ölschläger S. Neyts J. Günther S. (2011). Depletion of GTP pool is not the predominant mechanism by which ribavirin exerts its antiviral effect on Lassa virus. *Antiviral Res.* 91 89–93. 21616094 10.1016/j.antiviral.2011.05.006

[B82] Pfeiffer V. Papenfort K. Lucchini S. Hinton J. C. Vogel J. (2009). Coding sequence targeting by MicC RNA reveals bacterial mRNA silencing downstream of translational initiation. *Nat. Struct. Mol. Biol.* 16 840–846. 10.1038/nsmb.1631 19620966

[B83] Povolotsky T. L. Hengge R. (2012). ‘Life-style’ control networks in *Escherichia coli*: signaling by the second messenger c-di-GMP. *J. Biotechnol.* 160 10–16. 10.1016/j.jbiotec.2011.12.024 22226726

[B84] Pusic P. Sonnleitner E. Krennmayr B. Heitzinger D. A. Wolfinger M. T. Resch A. (2018). Harnessing metabolic regulation to increase Hfq-dependent antibiotic susceptibility in *Pseudomonas aeruginosa*. *Front. Microbiol.* 9:2709. 10.3389/fmicb.2018.02709 30473687 PMC6237836

[B85] Romeo T. Babitzke P. (2018). Global regulation by CsrA and its RNA antagonists. *Microbiol. Spectr.* 6. 10.1128/microbiolspec.RWR-0009-2017 29573256 PMC5868435

[B86] Römling U. Galperin M. Y. Gomelsky M. (2013). Cyclic di-GMP: the first 25 years of a universal bacterial second messenger. *Microbiol. Mol. Biol. Rev.* 77 1–52. 10.1128/MMBR.00043-12 23471616 PMC3591986

[B87] Salvail H. Caron M. P. Bélanger J. Massé E. (2013). Antagonistic functions between the RNA chaperone Hfq and an sRNA regulate sensitivity to the antibiotic colicin. *EMBO J.* 32 2764–2778. 10.1038/emboj.2013.205 24065131 PMC3801439

[B88] Santiago-Frangos A. Woodson S. A. (2018). Hfq chaperone brings speed dating to bacterial sRNA. *Wiley Interdiscip. Rev. RNA* 9:e1475. 10.1002/wrna.1475 29633565 PMC6002925

[B89] Sauer E. Schmidt S. Weichenrieder O. (2012). Small RNA binding to the lateral surface of Hfq hexamers and structural rearrangements upon mRNA target recognition. *Proc. Natl. Acad. Sci. U. S. A.* 109 9396–9401. 10.1073/pnas.1202521109 22645344 PMC3386104

[B90] Schirmer T. Jenal U. (2009). Structural and mechanistic determinants of c-di-GMP signalling. *Nat. Rev. Microbiol.* 7 724–735. 10.1038/nrmicro2203 19756011

[B91] Schmid J. Rühmann B. Sieber V. Romero-Jiménez L. Sanjuán J. Pérez-Mendoza D. (2018). Screening of c-di-GMP-regulated exopolysaccharides in host interacting bacteria. *Methods Mol. Biol.* 1734 263–275. 10.1007/978-1-4939-7604-1_21 29288461

[B92] Sharma I. M. Korman A. Woodson S. A. (2018). The Hfq chaperone helps the ribosome mature. *EMBO J.* 37:e99616. 10.15252/embj.201899616 29764978 PMC5983180

[B93] Sittka A. Lucchini S. Papenfort K. Sharma C. M. Rolle K. Binnewies T. T. (2008). Deep sequencing analysis of small noncoding RNA and mRNA targets of the global post-transcriptional regulator. *Hfq. PLoS Genet.* 4:e1000163. 10.1371/journal.pgen.1000163 PMC2515195 18725932

[B94] Sledjeski D. D. Whitman C. Zhang A. (2001). Hfq is necessary for regulation by the untranslated RNA DsrA. *J. Bacteriol.* 183 1997–2005. 10.1128/JB.183.6.1997-2005.2001 11222598 PMC95095

[B95] Smith K. D. Strobel S. A. (2011). Interactions of the c-di-GMP riboswitch with its second messenger ligand. *Biochem. Soc. Trans.* 39 647–651. 10.1042/BST0390647 21428955 PMC3689584

[B96] Sonnleitner E. Bläsi U. (2014). Regulation of Hfq by the RNA CrcZ in *Pseudomonas aeruginosa* carbon catabolite repression. *PLoS Genet.* 10:e1004440. 10.1371/journal.pgen.1004440 24945892 PMC4063720

[B97] Sonnleitner E. Hagens S. Rosenau F. Wilhelm S. Habel A. Jäger K. E. (2003). Reduced virulence of a hfq mutant of *Pseudomonas aeruginosa* O1. *Microb. Pathog.* 35 217–228. 10.1016/s0882-4010(03)00149-9 14521880

[B98] Sonnleitner E. Wulf A. Campagne S. Pei X. Y. Wolfinger M. T. Forlani G. (2018). Interplay between the catabolite repression control protein Crc, Hfq and RNA in Hfq-dependent translational regulation in *Pseudomonas aeruginosa*. *Nucleic Acids Res.* 46 1470–1485. 10.1093/nar/gkx1245 29244160 PMC5815094

[B99] Suchanek V. M. Esteban-López M. Colin R. Besharova O. Fritz K. Sourjik V. (2020). Chemotaxis and cyclic-di-GMP signaling control surface attachment of *Escherichia coli*. *Mol. Microbiol.* 113 728–739. 10.1111/mmi.14438 31793092

[B100] Tang Q. Yin K. Qian H. Zhao Y. Wang W. Chou S. H. (2016). Cyclic di-GMP contributes to adaption and virulence of *Bacillus thuringiensis* through a riboswitch-regulated collagen adhesion protein. *Sci. Rep.* 6:28807. 10.1038/srep28807 27381437 PMC4933901

[B101] Thomason M. K. Fontaine F. De Lay N. Storz G. (2012). A small RNA that regulates motility and biofilm formation in response to changes in nutrient availability in *Escherichia coli*. *Mol. Microbiol.* 84 17–35. 10.1111/j.1365-2958.2012.07965.x 22289118 PMC3312966

[B102] Tischler A. D. Camilli A. (2004). Cyclic diguanylate (c-di-GMP) regulates *Vibrio cholerae* biofilm formation. *Mol. Microbiol.* 53 857–869. 10.1111/j.1365-2958.2004.04155.x 15255898 PMC2790424

[B103] Tischler A. D. Camilli A. (2005). Cyclic diguanylate regulates *Vibrio cholerae* virulence gene expression. *Infect. Immun.* 73 5873–5882. 16113306 10.1128/IAI.73.9.5873-5882.2005 PMC1231145

[B104] Tuckerman J. R. Gonzalez G. Gilles-Gonzalez M. A. (2011). Cyclic di-GMP activation of polynucleotide phosphorylase signal-dependent RNA processing. *J. Mol. Biol.* 407 633–639. 10.1016/j.jmb.2011.02.019 21320509

[B105] Updegrove T. B. Correia J. J. Galletto R. Bujalowski W. Wartell R. M. (2010). E. coli DNA associated with isolated Hfq interacts with Hfq’s distal surface and C-terminal domain. *Biochim. Biophys. Acta* 1799 588–596. 10.1016/j.bbagrm.2010.06.007 20619373 PMC3072145

[B106] Updegrove T. B. Zhang A. Storz G. (2016). Hfq: the flexible RNA matchmaker. *Curr. Opin. Microbiol.* 30 133–138. 10.1016/j.mib.2016.02.003 26907610 PMC4821791

[B107] Valentin-Hansen P. Eriksen M. Udesen C. (2004). The bacterial Sm-like protein Hfq: a key player in RNA transactions. *Mol. Microbiol.* 51 1525–1533. 10.1111/j.1365-2958.2003.03935.x 15009882

[B108] Valentini M. Filloux A. (2019). Multiple roles of c-di-GMP signaling in bacterial pathogenesis. *Annu. Rev. Microbiol.* 73 387–406. 10.1146/annurev-micro-020518-115555 31500536

[B109] Vecerek B. Moll I. Bläsi U. (2005). Translational autocontrol of the *Escherichia coli* hfq RNA chaperone gene. *RNA* 11 976–984. 10.1261/rna.2360205 15872186 PMC1370782

[B110] Vincent H. A. Henderson C. A. Ragan T. J. Garza-Garcia A. Cary P. D. Gowers D. M. (2012). Characterization of *Vibrio cholerae* Hfq provides novel insights into the role of the Hfq C-terminal region. *J. Mol. Biol.* 420 56–69. 10.1016/j.jmb.2012.03.028 22484176 PMC3477312

[B111] Vogel J. Luisi B. F. (2011). Hfq and its constellation of RNA. *Nat. Rev. Microbiol.* 9 578–589. 10.1038/nrmicro2615 21760622 PMC4615618

[B112] Vytvytska O. Jakobsen J. S. Balcunaite G. Andersen J. S. Baccarini M. von Gabain A. (1998). Host factor I, Hfq, binds to *Escherichia coli* ompA mRNA in a growth rate-dependent fashion and regulates its stability. *Proc. Natl. Acad. Sci. U. S. A.* 95 14118–14123. 10.1073/pnas.95.24.14118 9826663 PMC24336

[B113] Vytvytska O. Moll I. Kaberdin V. R. von Gabain A. Bläsi U. (2000). Hfq (HF1) stimulates ompA mRNA decay by interfering with ribosome binding. *Genes Dev.* 14 1109–1118. 10809669 PMC316587

[B114] Wang R. Wang F. He R. Zhang R. Yuan J. (2018). The second messenger c-di-GMP adjusts motility and promotes surface aggregation of bacteria. *Biophys. J.* 115 2242–2249. 10.1016/j.bpj.2018.10.020 30447993 PMC6289094

[B115] Wang Y. C. Chin K. H. Tu Z. L. He J. Jones C. J. Sanchez D. Z. (2016). Nucleotide binding by the widespread high-affinity cyclic di-GMP receptor MshEN domain. *Nat. Commun.* 7:12481. 10.1038/ncomms12481 27578558 PMC5013675

[B116] Wei Q. Leclercq S. Bhasme P. Xu A. Zhu B. Zhang Y. (2019). Diguanylate cyclases and phosphodiesterases required for basal-Level c-di-GMP in *Pseudomonas aeruginosa* as revealed by systematic phylogenetic and transcriptomic analyses. *Appl. Environ. Microbiol.* 85:e01194-19. 10.1128/AEM.01194-19 31444209 PMC6803301

[B117] Wu D. C. Zamorano-Sánchez D. Pagliai F. A. Park J. H. Floyd K. A. Lee C. K. (2020). Reciprocal c-di-GMP signaling: incomplete flagellum biogenesis triggers c-di-GMP signaling pathways that promote biofilm formation. *PLoS Genet.* 16:e1008703. 10.1371/journal.pgen.1008703 32176702 PMC7098655

[B118] Xu G. Han S. Huo C. Chin K. H. Chou S. H. Gomelsky M. (2018). Signaling specificity in the c-di-GMP-dependent network regulating antibiotic synthesis in Lysobacter. *Nucleic Acids Res.* 46 9276–9288. 10.1093/nar/gky803 30202891 PMC6182147

[B119] Xu K. Shen D. Han S. Chou S. H. Qian G. (2021). A non-flagellated, predatory soil bacterium reprograms a chemosensory system to control antifungal antibiotic production via cyclic di-GMP signaling. *Environ. Microbiol.* 23 878–892. 10.1111/1462-2920.15191 32779811

[B120] Xue D. Tian F. Yang F. Chen H. Yuan X. Yang C. H. (2018). Phosphodiesterase EdpX1 promotes Xanthomonas oryzae pv. oryzae virulence, exopolysaccharide production, and biofilm formation. *Appl. Environ. Microbiol.* 84:e01717-18. 10.1128/AEM.01717-18 30217836 PMC6210124

[B121] Yakhnin A. V. Baker C. S. Vakulskas C. A. Yakhnin H. Berezin I. Romeo T. (2013). CsrA activates flhDC expression by protecting flhDC mRNA from RNase E-mediated cleavage. *Mol. Microbiol.* 87 851–866. 10.1111/mmi.12136 23305111 PMC3567230

[B122] Yang F. Tian F. Chen H. Hutchins W. Yang C. H. He C. (2015). The Xanthomonas oryzae pv. oryzae PilZ domain proteins function differentially in cyclic di-GMP binding and regulation of virulence and motility. *Appl. Environ. Microbiol.* 81 4358–4367. 10.1128/AEM.04044-14 25911481 PMC4475898

[B123] Yang F. Xue D. Tian F. Hutchins W. Yang C. H. He C. (2019). Identification of c-di-GMP signaling components in Xanthomonas oryzae and their orthologs in Xanthomonads involved in regulation of bacterial virulence expression. *Front. Microbiol.* 10:1402. 10.3389/fmicb.2019.01402 31354637 PMC6637768

[B124] Yuan X. Zeng Q. Khokhani D. Tian F. Severin G. B. Waters C. M. (2019). A feed-forward signalling circuit controls bacterial virulence through linking cyclic di-GMP and two mechanistically distinct sRNAs. *ArcZ and RsmB. Environ. Microbiol.* 21 2755–2771. 10.1111/1462-2920.14603 30895662 PMC6677606

[B125] Zamorano-Sánchez D. Xian W. Lee C. K. Salinas M. Thongsomboon W. Cegelski L. (2019). Functional specialization in *Vibrio cholerae* diguanylate cyclases: distinct modes of motility suppression and c-di-GMP production. *mBio* 10:e00670-19. 10.1128/mBio.00670-19 PMC6479008 31015332

[B126] Zhang A. Wassarman K. M. Ortega J. Steven A. C. Storz G. (2002). The Sm-like Hfq protein increases OxyS RNA interaction with target mRNAs. *Mol. Cell* 9 11–22. 10.1016/s1097-2765(01)00437-3 11804582

[B127] Zhao G. Jin Z. Wang Y. Allewell N. M. Tuchman M. Shi D. (2013). Structure and function of *Escherichia coli* RimK, an ATP-grasp fold, L-glutamyl ligase enzyme. *Proteins* 81 1847–1854. 10.1002/prot.24311 23609986

[B128] Zhao X. Koestler B. J. Waters C. M. Hammer B. K. (2013). Post-transcriptional activation of a diguanylate cyclase by quorum sensing small RNAs promotes biofilm formation in *Vibrio cholerae*. *Mol. Microbiol.* 89 989–1002. 10.1111/mmi.12325 23841714 PMC3807870

[B129] Zhou H. Zheng C. Su J. Chen B. Fu Y. Xie Y. (2016). Characterization of a natural triple-tandem c-di-GMP riboswitch and application of the riboswitch-based dual-fluorescence reporter. *Sci. Rep.* 6:20871. 10.1038/srep20871 26892868 PMC4759541

